# Design of a Device for Lower Limb Prophylaxis and Exercise

**DOI:** 10.1109/JTEHM.2020.3037018

**Published:** 2020-11-09

**Authors:** K. Vinay, Krishna Nagaraj, H. R. Arvinda, V. Vikas, Madhav Rao

**Affiliations:** 1 Surgical and Assistive Robotics LaboratoryIIIT-BangaloreBengaluru560100India; 2 Department of NeuroradiologyNIMHANSBengaluru560029India; 3 Department of NeurosurgeryNIMHANSBengaluru560029India

**Keywords:** Blood flow, lower limb, prophylaxis, rehabilitation

## Abstract

The problem of immobility of legs leading to a potentially life threatening condition including deep venous thrombosis (DVT) is well known. The reduced mobility of leg affects a large number of patients in a wide range of clinical scenarios spanning from swelling of the legs to pulmonary embolism. In normal human beings, an elegant system of venous return, both active and passive is responsible for prevention of deep venous thrombosis. The paper proposes a prophylaxis and exercise device that mimics the natural principles of venous return to promote the blood flow. The device is based on electromechanical actuation, and simultaneous alternating compression mechanism, that is compact and, suitably form fitted in design and additionally requires no specialised training for the usage. The device was tested on a healthy volunteer on two different days and findings support the efficacy of the prophylaxis and exercise device in significantly improving the blood flow rate in the lower limb. The prototype device is considered as a major step towards designing a clinically validated lower limb device.

## I. Introduction

Approximately more than 73% of the thrombi are generated in the deep veins of the lower limb [1]. The prolonged immobilization either due to long hour sedated position of patient in a surgical theatre or due to age related bed ridden patient develops thrombosis in the deep veins of the leg [1]. The retained thrombi causes multiple effects. The thrombus can extend along the veins causing obstruction to the venous outflow of the leg. The problem manifests as swelling in the legs which can result in a secondary infection. This phenomenon in a chronic phase can result in venous ulcers in the leg which are non healing. One of the significant risk in the patients suffering from lower limb problems is the thrombus propagating and embolising. Depending on the size of the embolus, various symptoms and disease processes are triggered. Although Mild emboli are not symptomatic, frequent showers of emboli cause obstruction of pulmonary capillaries, resulting in breathlessness, and patients live on ventilatory support. The loss of pulmonary function due to the emboli may cause acute worsening of clinical conditions in such patients. Larger emboli occlude the larger pulmonary arteries. A large ‘saddle embolus’ straddles the pulmonary arteries and causes acute right heart failure [2]–[7]. The veins within the Soleus muscle, which are located in the deep venous system of the leg, are prone to venous stasis. Even in the supine position, when the pressure gradient to the right atrium is lesser, blood flow into the heart is passive. The Soleal pump is responsible for reducing vein capacitance in the standing position. This is achieved partly due to muscle tone in the calf muscles and the valves within the veins of the lower limb. Walking causes muscle activation which in turn exerts a force on the walls of the veins causing blood to be pumped from the legs to the heart [1]. In patients who are bed ridden, leg mobility is reduced. This causes decrease in the flow of blood within the deep veins. The dilatation of the veins increases capacitance which in turn reduces blood flow further. This predisposes to thrombosis. A major concern is that majority of these cases are asymptomatic. Patients develop symptoms often after full blown thrombosis occurs. A wide range of patients suffer from thrombosis related effects. Patients who have undergone surgery, orthopaedic procedures, suffer fractures in the lower limbs, patients with neuromuscular weakness are all susceptible to this group of disease [8]. 5% to 10% of passengers develop this syndrome in long haul flight journeys [9].

The lower limb prophylaxis and exercise is the focus of extensive research to control lower limb related problems suffered by many. Various modalities have evolved to overcome thrombosis issues especially arising from deep in the veins. Two distinct approaches are practised. One has been the pharmacological method of using anti-coagulation drugs. Another approach of non-pharmacological type includes mechanical compression either statically applied by using stockings or dynamic compression using a pneumatic pump, which cyclically compresses the calf. Electrical stimulation of muscle groups in the lower limb have also been tried [8]. Each of these techniques have shown benefit in reducing the incidence of DVT. However, commensurate with their efficacy, side effects are also noted. Thus, pharmacological means which are the gold standard of treatment have the highest risks, whereas mechanical methods which are relatively safe have a lower efficacy in preventing thrombosis. The optimal method of lower limb prophylaxis is a matter of debate and there is consensus that large scale randomised clinical trials are needed to evaluate the efficacy and risks of each of these techniques [10]–[12].

Previously a device is described in [13] wherein a pneumatically expandable bladder was used that would apply necessary forces on the body parts to achieve compression and hence increase blood flow. However, this device does not make use of the ankle movement to generate blood flow in the lower limb. In another proposed device [14] a mechanical contraption is realized to actuate the ankle to the required angle, however, the actuation mechanism is complex to wear and use. Another device described in [15] uses soft pneumatic robotics to achieve the actuators in order to move the ankle, however, had a limited range of actuation. The pneumatic compression and the ankle movement independently do increase blood flow, however when combined together the increase in blood flow is much higher [16]. The main cause of failure of lower limb prophylaxis devices was the complexity of use, and hence a reduced usage by the patients and caretakers [17]. A versatile, portable, easy to use and cost-effective device that could perform both the actuation’s is needed to face these challenges.

In this paper, a design of a device for lower limb prophylaxis and exercise has been proposed. A working prototype has been developed and applied on a healthy volunteer setting on two different occasions with blood flow measurement results observed using a medical grade ultrasonography device. The device trial was performed by reporting improvement in blood flow in the venous system of the lower limb on using the proposed device. In the earlier work [18], a mechanism to automate the ankle actuation, and compression of calf muscles and correlation with heart rate-$SPO_{2}$ [19] signal had been described. In this work, the prophylaxis device is synchronised with respiration rate, which is more medically accepted, for device activation. The changes in deep venous flow was evaluated by ultrasonography device measurements obtained by an experienced radiologist and measured while the device was in operation. The results were analysed to showcase the utility of the device for the future clinical usage and possibly as an independent domestic device. The device in conjunction with ultrasonography doppler device is potential setup for the future clinical and surgical wards for maintaining adequate blood flow, deep in the veins of the lower limb.

## II. Proposed Design

**FIGURE 1.**
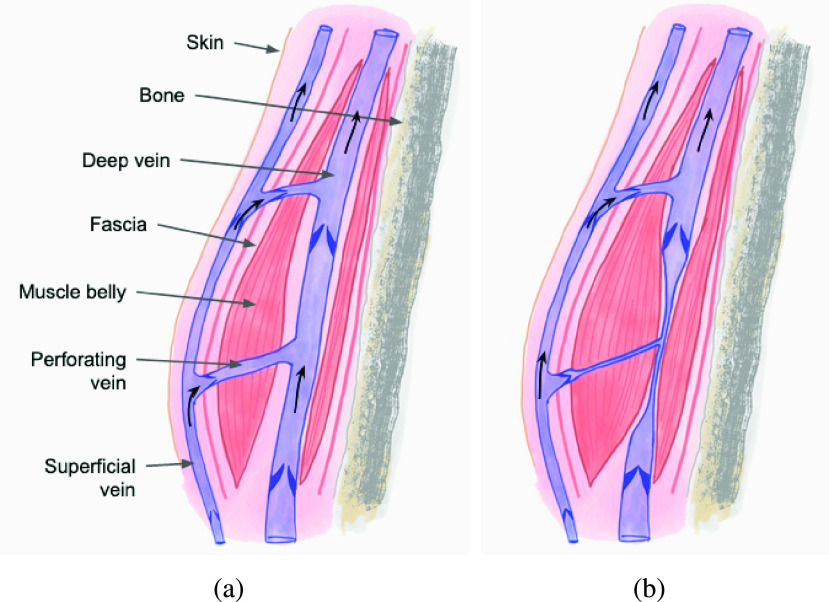
A picture redrawn from [1] showing blood flow in the deep venous system under (a) relaxed muscle state, and (b) under inflated state.

The device is designed to assist the blood flow in a natural mechanism to improve venous outflow. As shown in the Figure 1, the deep veins that allows the flow of blood is surrounded by calf muscles [1]. The deep calf vein primarily receives the blood flow from foot sole [20]. The superficial system is situated outside of the muscles of the leg. The perforators are conducting vessels which connect the deep and superficial venous systems through muscles. In the normal state, when the calf muscles, chiefly the Soleus muscle contracts, the deep veins are compressed which results in flow of blood in these veins towards the heart. The presence of valves within these veins prevent backflow of blood during muscle relaxation. Within the superficial system, blood flows towards the heart due to the negative pressure within the right atrium. Also, during the phase of Soleus muscle relaxation, blood enters the deep venous system due to the differential pressure between the superficial and deep venous system. The mechanism has been termed as the Soleal pump or the second heart, indicating the importance of the mechanism in delivering deoxygenated blood from the lower limb back to the heart. The proposed design aims to replicate the mechanism of the soleal pump. Flexion and extension at the ankle would result in change in length of the Soleus, which in turn is expected to change pressure within the deep venous system resulting in pumping of blood similar to the natural mechanism. The added component of compression synchronised with the ankle movement is meant to improve the efficacy of the mechanism [4].

**FIGURE 2.**
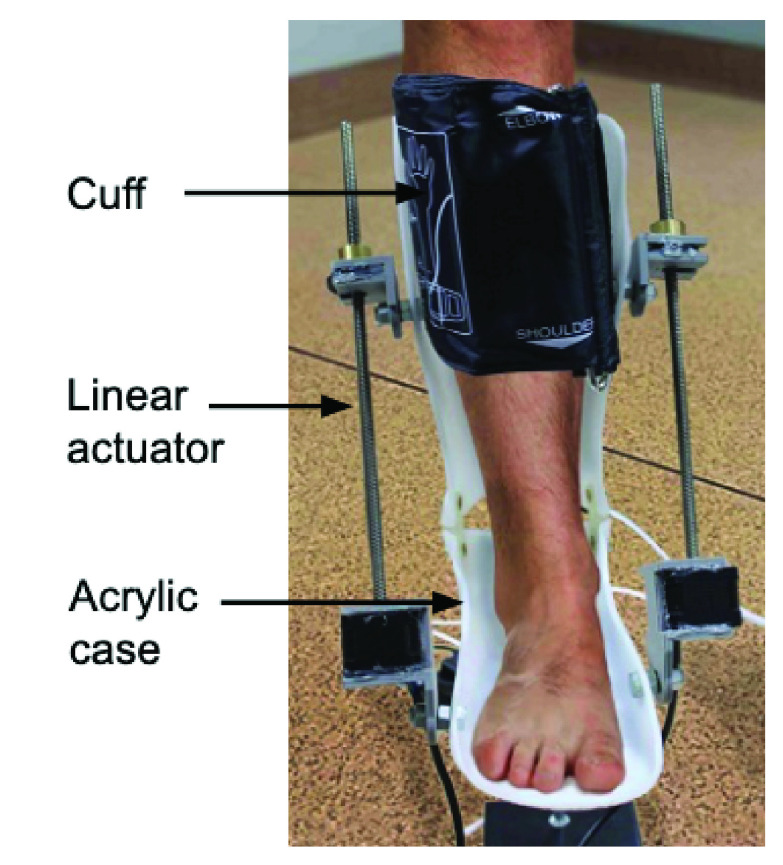
A picture of the prophylaxis device showing linear actuator and an inflatable cuff wrapped around calf muscles of lower limb.

The mechanism was implemented by actuating a standard ankle foot orthotic with only one degree of freedom. The orthotic was customised to have restricted movement to mimic ankle dorsiflexion and plantar flexion. A pair of linear stepper actuators were used to generate movement of ankle. The actuators are fixed on the sides of the customised orthotic. The stepper motor is activated to provide flexion and extension movement for the legs. A motor driver A3967 IC [21] each is interfaced to the stepper motor to supply adequate current to perform ankle movement. The device was configured to run on two actuation speeds and was designed to operate either in autonomous or manual mode. The autonomous mode is designed for unconscious or sedated patients, whereas the manual mode is targeted towards conscious patients with restricted movement, and is designed to be self-operated with minimal assistance. A balloon type rubber cuff to compress calf muscles along with foot actuation is included in the current design. The cuff inflation and deflation is electrically controlled. A snapshot of the device showing a pair of linear actuators assembled on the orthotic device along with the inflatable cuff wrapped around the legs of a subject is shown in the Figure 2.

**FIGURE 3.**
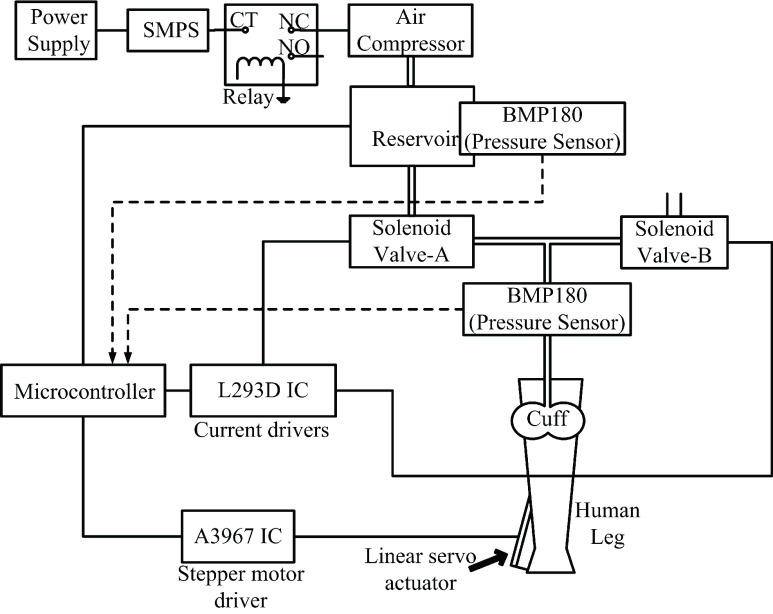
System designed for lower limb prophylaxis device.

For cuff inflation, a reservoir maintaining a pressure of 2200 mbar is designed which is controlled by an air compressor of 12V, and 15A current rating. A switched mode power supply (SMPS) is required to operate the rated air compressor. The prefilled reservoir supplies air to the rubber cuff via a solenoid valve (valve-A) to impart pressure on the calf region of the lower limb. Another solenoid valve (valve-B) is placed forming a T joint with the former valve to alternately release the pressure built in the cuff and relax the calf muscles. A pressure of 1100 mbar in the cuff is maintained during compression activity and atmospheric pressure is realized during deflation phase. The two valves: A and B are sequentially opened and closed to impart alternating compressing forces on the calf muscles. In addition, a sensor is placed in the reservoir to indicate any fall in the reservoir pressure below 2200 mbar, and restore air inside the reservoir by turning on the air compressor pump. L239D [22] motor drivers are interfaced to the solenoid valves, to supply adequate current for operating valves. The overall system architecture with T-joint valves and pressure sensors installed at the outlet of reservoir and close to the rubber cuff are shown in the Figure 3.

**FIGURE 4.**
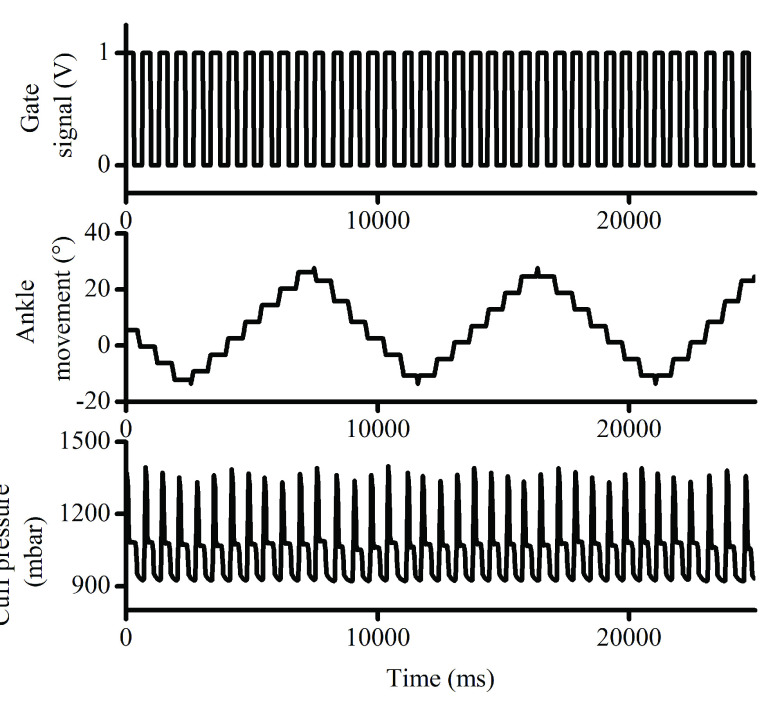
Graph showing device operation with a generated gate signal to initiate lower limb ankle actuation, and compression cycle for calf muscles.

The proposed design required simultaneous operation of ankle movement and calf compression as a method to optimise blood return from the leg. A timed mechanism to actuate ankle, and cuff compression-relaxation cycle, triggered by a reference signal was implemented as shown in the Figure 4. The improvement in blood flow rate is recommended to be instantiated along the breathing pattern of the patient to correlate with pulmonary vascular compliance. Blood flow and cardiac filling vary with respiratory cycles. Thus, the device was synchronised to physiological blood flow characteristics. The inhalation and exhalation phases of the breathing signal are further quantized to binary states, and utilized as input gate signal to generate timed ankle actuation, and calf compression cycles. The gate signal depicted in the Figure 4 indicates the operation of device for a reference of 1.4 Hz, which is considered an extreme case of breathing.

The device is designed for the following timed sub-tasks.

• The ankle movement during the high level of the gate signal representing inhalation event, induces continuous venous blood flow, and
• the additional compressing force applied on the calf muscles during the high level of the gate signal aids in pumping blood from the superficial veins to the deep veins and reduce deep venous compliance.

The device can achieve a maximum cuff pressure of 4000 mbar range but is limited to 1400 mbar for safety reasons. Figure 4 demonstrates that a pressure of 1100 mbar is achieved during the positive level of gate signal. The compressive forces are applied to calf muscle during the high level of the gate signal, representing inhalation event for the subject under treatment. The high level of the gate signal opens the valve-A and closes valve-B, thereby filling the cuff from the reservoir. The sudden rise in the cuff pressure reading as shown in the Figure 4 is attributed to the instantaneous filling of air inside the wrapped cuff, however, the steady state pressure of 1100 mbar is maintained post-transient. During the low level of the gate signal, the valve-B is opened, and valve-A is closed to release air from the cuff and reduce pressure of the cuff to atmospheric level. The venting process is considered time consuming as against filling of air through a prefilled reservoir. In the high level of the gate signal, the designed closed loop system ensures that the pressure built in the inflatable cuff is restored and maintained at 1100 mbar, whereas at the low level of the gate signal, atmospheric pressure is achieved.

The generated high level gate signal also initiates linear stepper motor to move the ankle joint. In low level of gate signal, the ankle is held stable, without any movement. The movement of the ankle leading to dorsoflexion and plantarflexion [23] is expressed in terms of the angle formed between the leg and foot. The range of motion for ankle extension and flexion is programmable and can be set for individual patients under the supervision of the treating physician. In general, the device was highly reproducible for both ankle motion, and calf compression, when used as shown in the table 1. The plantarflexion and dorsiflexion due to motor movements are reproducible with ± 0.015°, whereas the applied cuff inflated pressure showed an accuracy of ±0.02 mbar. The other details related to the lower limb device are stated in the table 1.

**TABLE 1:** Device Characteristics

Parameters		Dimensions
Size of Platform		25 cm $\times11$ cm
Weight of the Setup		1.265 Kg
General range	Plantarflexion	27.6°
	Dorsiflexion	−13.6 °
	Cuff inflation	1013 to 1400 mbar
Maximum range	Plantarflexion	44.5°
	Dorsiflexion	−30.0 °
	Cuff inflation	1013 to 4000 mbar
Accuracy	Ankle Reproduction	0.015°
	Cuff inflation	0.02 mbar

The flexion and extension of lower limb foot, occurs in discrete step of 6° during the high level of the gate signal as shown in the Figure 4. The linear stepper motor used in the prototype device achieves a maximum of 44.5 ° in frontal flexion, and −30 ° along dorsoflexion, The general range of 40 ° of ankle movement including flexion and extension is covered in 12 cycles of the reference gate signal. In the low level of the gate signal, the ankle position is held constant with no changes, to avoid any discomfort for patients, by continuously moving the ankle. Constant actuation of the linear stepper motor is also possible which will cover the range of 40 ° in 1 gate cycle, however, a discrete step of ankle positioning is adequate to prevent blood pooling.

## III. Experimental Results

**FIGURE 5.**
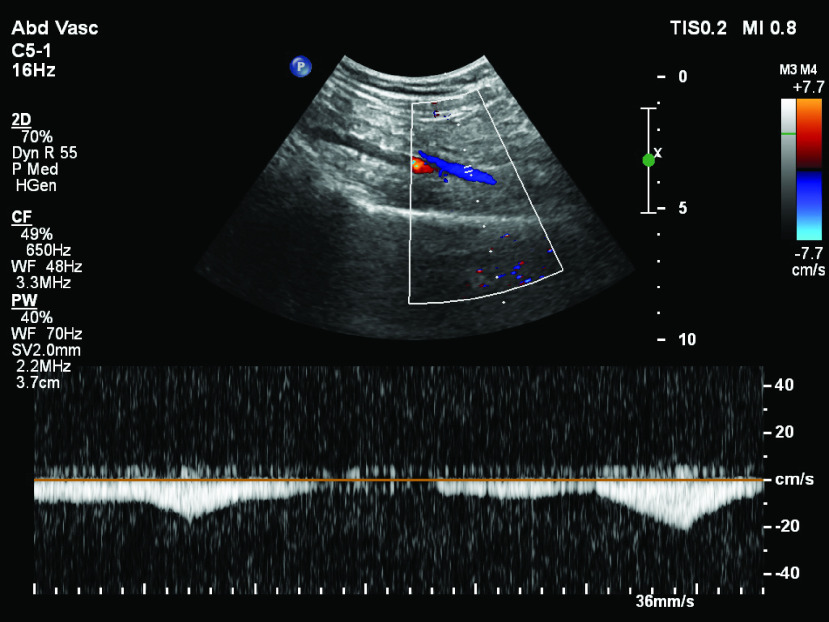
An image acquired from ultrasonography device while scanning the popliteal fossa region of the subject.

The prototype device was applied on a healthy subject twice on two different days in a gap of two months to ensure consistency of results over time. A Doppler ultrasonography device (Philips EPIQ 7 [24]) was simultaneously utilized on the subject under investigation to report any changes in blood flow. The ultrasound probe was placed above the vein of the popliteal fosa region to measure the blood flow in the back of the knee joint. The velocity gradient displayed on the screen was observed to infer the locations of veins and arteries. One such snapshot of the ultrasound scanned image is shown in Figure 5. The device recorded the blood flow rate that was observed in the units of centimeters per second. The area of interest where blood flow measurement is intended, was entered in the ultrasonography device. The device recorded the blood velocity of the selected vein region in a buffer of fixed duration. Screen shots of subsequent buffers were captured and transferred to a computer, where the images were manually stitched and blood flow rate data was extracted.

**FIGURE 6.**
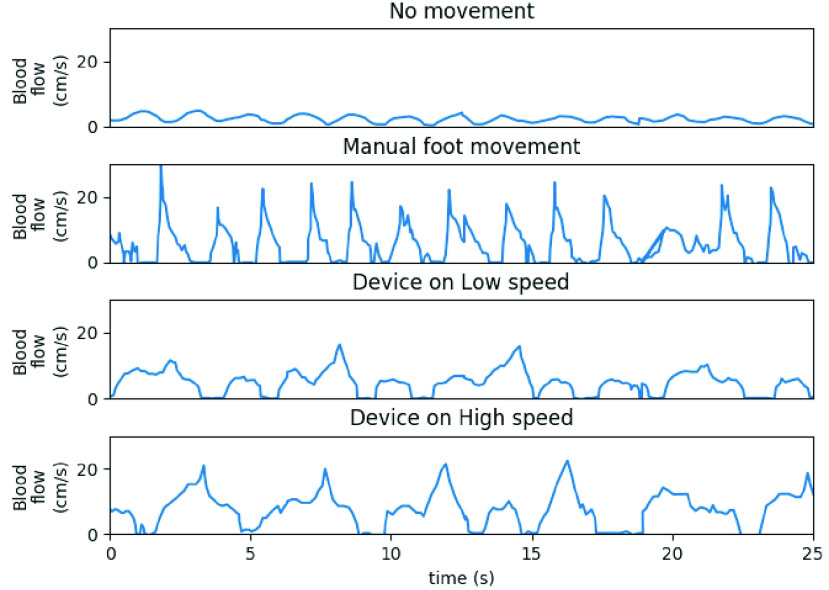
Measurement of blood flow with prophylaxis device operated with ankle actuation at different speeds.

For the first experimental outcome, the subject was rested in a right lateral recumbent position with leg mildly abducted and laterally rotated so that the ultrasound probe may be placed on the popliteal fossa region. The subject was asked to relax for 15 minutes before the experiment was initiated. The device was applied on the right leg of the subject and the device was programmed to operate in two different modes: high speed, and slow speed mode. In the high speed mode, the ankle was moved in either direction at a speed of 20°/sec, and low speed mode actuates the ankle at 10°/sec speed. Before actuating the device, the blood flow of the subject was measured with Doppler ultrasonography device without any movement, and with passive ankle movement. The popliteal fossa region in right leg of the subject under trial was simultaneously scanned with doppler ultrasonograpy device to measure blood flow in the popliteal veins. In this experiment, the cuff operation was disabled, to see assisted ankle actuation effect on the blood flow rate. The subject was asked to breathe at a consistent, and comfortable rate. The recorded breathing rate was observed to be 30 per minute, which was slightly high when compared to the breathing rate at rest. The higher than average breathing rate [25] was associated with mild exercise strain [26]. This breathing rate also allowed the ultrasound device to record more cycles of respiration without loss in resolution whilst utilizing the maximum buffer memory available in the ultrasonography device. The low speed and high speed assisted ankle actuation induced blood flow rate, were further compared with flow rates measured with no movement and voluntary ankle movement events. Figure 6 indicated higher blood flow rate for assisted ankle actuation when compared with either no movement or passive self ankle movement. It is clearly seen that the blood flow rate was significantly high when the device was operated at high speed of ankle actuation. Table 2 reports the average blood flow rate from the measured profile. Note that, in this experiment, device operation was not synchronised with breathing. The assisted ankle actuation at high speed showed an improvement of 209% in blood flow rate, when compared to the ankle at rest. The slow speed actuation configuration shows an improvement of at least 98% when compared to no movement. The findings report a significant increase in the deep venous outflow of the leg. The proposed device along with a medical grade ultrasonogrpahy device is useful for a wide range of clinical settings ranging from orthopaedic patients, patients undergoing long duration surgery and even in patients undergoing neuromuscular rehabilitation.

**TABLE 2:** Blood Flow Rate Measured by the Doppler Ultrasonography Device on a Subject in Different Conditions

Conditions	Average blood flow rate (cm/s)
No movement	2.36
Self movement	5.73
Assisted ankle actuation at slow speed	4.68
Assisted ankle actuation at high speed	7.31

A second experiment on the same volunteer post 2 months was conducted as follows. The prophylaxis device was operated in synchronization with the breathing pattern of the healthy volunteer. The subject was given a switch to press while inhaling and release on exhaling to generate a reference gate signal. The reference signal was used to synchronize ankle actuation mechanism and alternating cuff compression cycles. The manual switching activity was preferred over mechanical strain sensor or chest strap to avoid any physical discomfort for subject when in the right lateral recumbent position for measuring blood flow rate. The ankle actuation configured at high speed of 20°/sec, and cuff inflation mode was operated separately in this experiment and the blood flow rate was measured using Ultrasonography device. In addition, the device was operated with ankle actuation, and cuff compression simultaneously, and resultant blood flow was measured.

**FIGURE 7.**
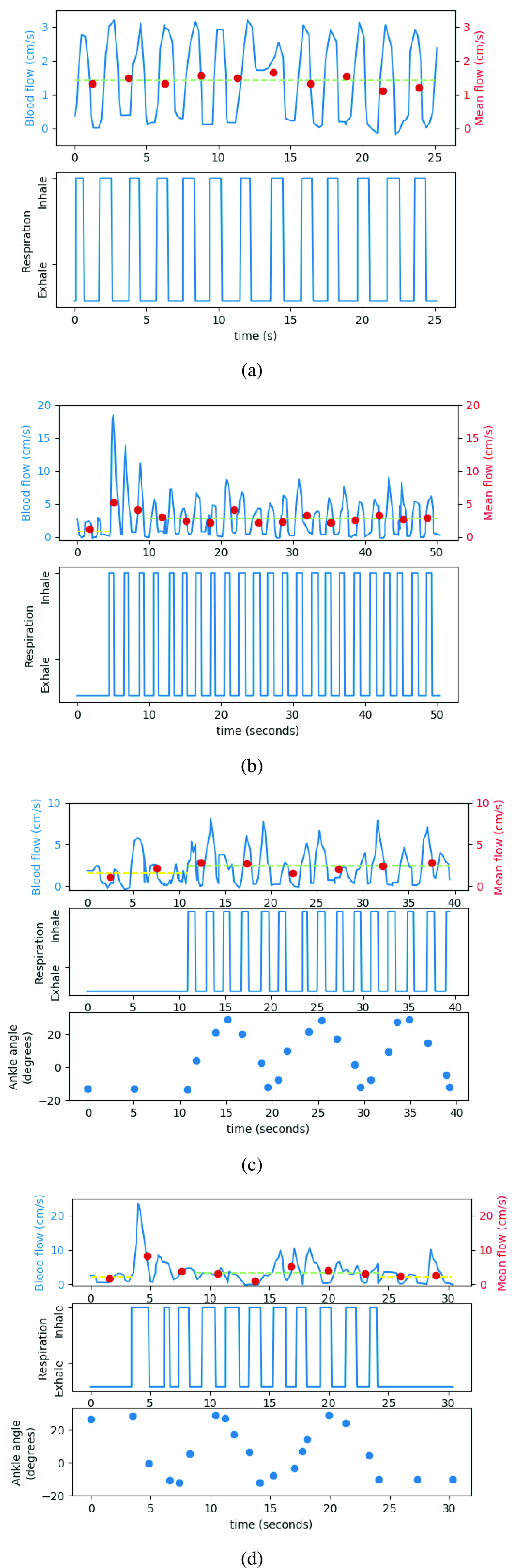
Blood flow measurement using Doppler ultrasound device for the subject when treated with (a) No movement, (b) alternating calf compression, (c) ankle actuation, and (d) ankle actuation and alternating calf compression mechanism.

Figure 7 shows the blood flow rate recorded by Ultrasonography device for all the four modes of prophylaxis device operation including no movement by the subject. The measured blood flow rate over four modes of operation suggests that concurrent operation of ankle actuation and calf compression, yields significant improvement in the blood flow rate when compared to no movements, and other individual modes of operation. In the figure 7, average blood flow rate for a constant window is also included in the form of dotted points to do comparative analysis. An average blood flow rate for entire duration in steady state while the device is operating and not operating is also included in each part of the Figure 7 in the form of dotted straight lines to effectively see the improvement in blood flow rate when the device is in operation. The standalone alternating compression and relaxation cycles applied on calf muscles improves the blood flow rate when compared to no movement, as shown in Figure 7(b), however the blood flow rate drops to a steady rate post the initial rush of blood from superficial veins. Similar results of calf muscles compression through pneumatic pumping were observed in [1].

The alternating compressing cycle applied on the calf muscles pushes the pooled blood in the deep venous system initially, whereas the subsequent contractions of muscles does not have the same amount of blood, hence the alternating compression on calf muscles is not considered effective for critical patients. However the improvement in blood flow is considered suitable for passengers travelling in long flights with no prior medical problems. The independent assisted ankle actuation improves the blood flow rate and presents similar improved steadied blood flow rate when compared to calf muscles compression, as shown in the Figure 7(c). The ankle actuation primarily triggers blood from foot to pass through deep veins.

Both mechanism including assisted ankle actuation and alternating compression cycles when applied on the lower limb shows the highest improvement in the blood flow rate over other individual modes of operation, as shown in Figure 7 (d). The table 3 shows peak value of blood flow rate and mean blood flow rate post initial rising cycles, as derived from the measurements reported in figure 7 for all four modes of operation. The table 3 emphasizes the same with quantified data suggesting that the peak blood rate, and average steady state blood rate improves significantly when both ankle actuation and compression mode is concurrently operated. The average blood rate level induced by individual ankle actuation, and calf compression is similar, however the combined effect of assisted ankle actuation and calf compression presents significant improvement. The continuous movement of foot due to assisted ankle actuation draws new volume of blood from foot to deep veins and superficial veins. The simultaneous operation of compressing forces applied on calf muscles drives the blood flow from superficial veins to deep veins, thereby supplementing the assisted ankle actuation mechanism and obtaining an improved blood flow rate. The dual mechanism of our device is unique and is expected to improve the blood flow in the lower limb and possibly make a difference in the field of prophylaxis and exercise of the lower limb.

**TABLE 3:** Blood Flow Rate Measured by Doppler Ultrasonography Device on a Subject Under Different Conditions

Conditions	Blood flow rate (cm/s)	
	Peak	Average rate in Steady state
No movement	3.1	1.42
Assisted ankle actuation	8	2.44
Calf compression	18	2.71
Assisted ankle actuation and calf compression	22	3.30

## IV. Conclusion

Thrombosis in the deep veins of the leg is caused due to an alteration in the physiology. Normal movement of the leg when impaired, causes complex changes in the muscular and vascular compartments of the leg causing thrombosis and subsequently embolism. There is no single technique either pharmacological or mechanical which is an optimal solution to the problem. The device presented in this work attempts to mimic the natural mechanism to the fullest. The device is an example of a continuous passive leg motion and has been modified for prophylaxis and exercise. The design is compact and is meant to serve a wide range of patients ranging from the intra-operative setting, orthopaedic patients, patients suffering from neuromuscular disability and stroke. Experimental verification of the hypothesis is presented to demonstrate a significant improvement in blood flow on using the device on a healthy subject on two different days. The individual components viz. external compression and ankle actuation individually have been demonstrated to improve blood flow. The improvement in blood flow is demonstrated through the increased blood flow reported in the popliteal veins which is the dominant vein from the leg. The two mechanisms in the form of calf compression and ankle actuation attempt to mimic the natural mechanisms which improve the blood flow. The proposed lower limb prophylaxis and exercise device is seen as a step towards developing a clinically approved device, which is considered significant in the context of vast patient population having lower limb mobility issues.
